# Quantitative Characterization of Elemental Segregation in Inconel 718 Superalloy by Micro-Beam X-ray Fluorescence Spectroscopy and Its Correlation Study

**DOI:** 10.3390/ma16227163

**Published:** 2023-11-14

**Authors:** Xuefan Zhou, Dongling Li, Qingqing Zhou, Fan Jiang, Yan Song, Wanying Liang, Mingbo Liu, Xuejing Shen, Haizhou Wang

**Affiliations:** 1 Beijing Advanced Innovation Centre for Materials Genome Engineering, Central Iron and Steel Research Institution, Beijing 100081, China; zhouxuefan21@mails.ucas.ac.cn (X.Z.); zhouqingqing@ncschina.com (Q.Z.); jiangfan20@mails.ucas.ac.cn (F.J.); songyan21@mails.ucas.ac.cn (Y.S.); liangwanying2023@163.com (W.L.); liumingbo@ncschina.com (M.L.); wanghaizhou@ncschina.com (H.W.); 2 Beijing Key Laboratory of Metal Materials Characterization, The NCS Testing Technology Co., Ltd., Beijing 100081, China; shenxuejing@ncschina.com; 3 Beijing Advanced Innovation Center for Materials Genome Engineering, National Center for Materials Service Safety, University of Science and Technology Beijing, Beijing 100083, China

**Keywords:** Inconel 718 superalloy, micro-beam X-ray fluorescence, elemental segregation, scanning electron microscope, Nb-containing precipitate

## Abstract

Inconel 718 (IN718) nickel-based superalloy is widely used in aerospace and nuclear applications owing to its excellent comprehensive mechanical properties, oxidation resistance, and hot corrosion resistance. However, the elemental segregation caused by heterogeneous solidification during casting has great influence on the mechanical properties. Therefore, accurately characterizing the segregation behavior is necessary. Traditional quantitative characterization of elemental segregation uses various sampling methods, in which only macroscopic segregation results are obtained. In this study, micro-beam X-ray fluorescence (μ-XRF) is used for the quantitative characterization of element micro-segregation in IN718 superalloy. The concentration distributions of Cr, Fe, Mo, Nb, and Ti in IN718 alloy are determined with optimized testing parameters, and the degree of elemental segregation in different regions of the analytical area is calculated. It is found that the segregation degree of Nb and Ti in the testing area is larger than other alloying elements. The correlation between the microstructure distribution and the segregation degree of Nb and Ti has been studied using scanning electron microscopy (SEM) combined with energy-dispersive spectrometry (EDS). There is severe segregation of Nb and Ti in areas where Nb-containing precipitates are accumulated. The distribution of abnormal signals of Nb with a high fluorescence intensity has a close relationship with the area of precipitates-enriched Nb.

## 1. Introduction

Inconel 718 superalloy (IN718) has excellent oxidation resistance, mechanical properties, and fatigue and creep properties at high temperatures, which makes it widely used in aerospace, oil, and other industries [1,2,3,4]; however, there are complex interactions among elements in multi-alloy systems. A large number of alloying elements, such as Al, Ti, Nb, and Mo, in IN718 superalloy can lead to a strong tendency toward micro-segregation. During heterogeneous solidification, these elements become enriched in the precipitated phase, such as the Laves phase, which reduces the effect of solution strengthening. Laves phase often peels off the matrix under tensile force, and melted Laves phase can form micro-cracks [5,6,7,8]. In addition, severe micro-segregation results in macro-segregation, even forming “white spots”, which can seriously damage the mechanical properties of the material and lead to the scrapping of products. Therefore, quantitatively characterizing the elemental segregation is important for superalloys to evaluate the material quality and improve the mechanical properties [9,10,11,12].

Traditional methods for macro-segregation characterization of alloys include sulfur imprinting, metallographic microscopy, chemical analysis, and original position statistical distribution analysis [13,14,15,16]. Metallographic microscopy and sulfur printing can only qualitatively characterize the segregation of carbon or sulfur, while chemical analysis acquires quantitative results by drilling and sampling from alloys with complicated testing steps. Original position statistical distribution analysis of metal materials is usually based on spark-source atomic-emission spectroscopy (AES), laser-induced breakdown spectroscopy (LIBS), or laser-ablation inductively coupled plasma mass spectrometry (LA-ICPMS). Hitherto, it has been applied to quantitative analysis of elemental segregation in materials such as low-alloy steel, additive manufacturing stainless steel, and nickel-based superalloys [17,18,19,20,21]. However, these traditional characterization methods are destructive, and their spatial resolution needs to be improved. Although the electron probe microanalysis technique (EPMA) has high spatial resolution for element micro-segregation characterization and the morphology of microstructures can be simultaneously obtained, the sample preparation requires great caution, and the observation area is limited. All these characterization methods cannot meet the demands of non-destructive, efficient, and cross-scale identification of element segregation for different alloys.

In recent years, micro-beam X-ray fluorescence spectroscopy (μ-XRF) has shown obvious advantages in the analysis of elemental distribution. It uses different types of targets as light sources, and the X-ray focused through a multi-channel capillary generates a smaller spot size, which greatly enhances the light intensity and improves the peak-to-background ratio (PBR), thus realizing non-destructive and high-resolution elemental analysis [22,23,24,25]. Simple sample preparation, high resolution, and the non-destructive nature of the analysis provide strong advantages for μ-XRF in the characterization of element distributions of alloys, geological minerals, and biological samples [26,27,28,29,30,31,32,33,34]. For example, μ-XRF is used to characterize the distribution of sulfur in stalagmite samples [35]. In terms of alloys, μ-XRF has been used to determine the elemental distributions of T4-15 aluminum alloy, and segregation zones of Al, Cr, and Zr in the central layer have been identified [36]. However, μ-XRF has not yet been established as a mature method for quantitative characterization of element segregation in superalloys owing to the matrix effect caused by complex elements. The test conditions and quantitative methods of μ-XRF for different complex systems need to be optimized as well. Moreover, the causes of segregation still need further investigation.

The aim of this research was to investigate a method of detecting elemental segregation in IN718 superalloys using μ-XRF. Scanning electron microscopy with energy-dispersive spectroscopy (SEM-EDS) was applied to characterize the Nb-containing precipitates distributed in the whole testing area, and the correlation between elemental segregation and microstructure distribution was discussed.

## 2. Materials and Methods

A piece of IN718 superalloy numbered S1 was used. It was produced by a triple process of vacuum induction melting, vacuum arc remelting, and electro-slag remelting, then subjected to solution and aging treatments. The chemical composition of S1 is shown in Table 1.

**Figure 1:**
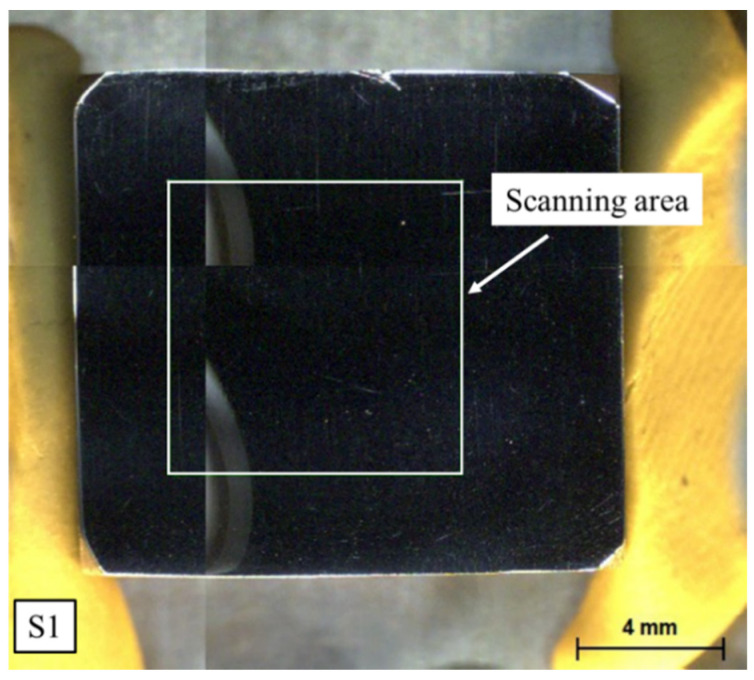
Scanning area of S1.

The S1 sample was ground using sandpaper of various mesh sizes and polished using 3.0 μm alumina polishing paste until a bright mirror surface was achieved. A rectangular test area of 8 mm × 8 mm was marked. The scanning area is shown in Figure 1.

To quantitatively characterize the distribution of different elements by μ-XRF, 11 nickel-based alloy spectrometric reference materials were selected to obtain calibration curves. The chemical compositions of these reference materials are shown in Table 2.

**Figure 2:**
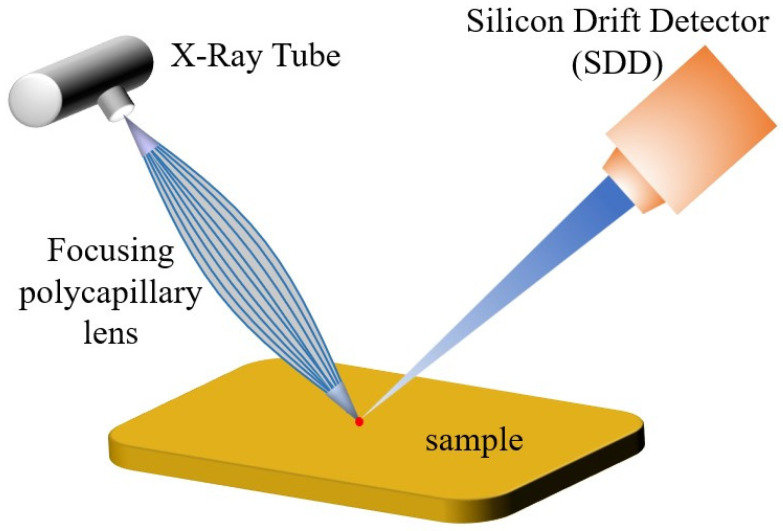
Schematic of the μ-XRF setup [37].

A micro-beam X-ray fluorescence spectrometer (M4 TORANDO, Bruker Corporation, Mannheim, Germany) was used to analyze the elemental distribution on the S1 test area. This instrument uses a rhodium target as a light source and has an X-ray tube with a maximum power of 30 W. The maximum tube voltage is 50 kV, and the tube current can reach 600 μA. Two X-flash silicon drift detectors were used, which can achieve an energy resolution of 145 eV for the Mn-Kα line. A poly-capillary X-ray optics, having a cone angle of 34°, were used to focus the non-polarized Bremsstrahlung at the surfaced of the sample, which enabled a high spatial resolution of approximately 20 μm, as shown in Figure 2. The vacuum system is equipped with a vacuum pump that can achieve 2000 Pa inside the instrument. In this work, the main elements were quantified using the Kα line system, with energies of *E*_Cr_ = 5415 eV, *E*_Fe_ = 6405 eV, *E*_Mo_ = 17,480 eV, *E*_Nb_ = 16,615 eV, *E*_Ni_ = 7480 eV, and *E*_Ti_ = 4512 eV.

The distribution of Nb precipitates in the same testing area was characterized using SEM-EDS (VEGA3, TESCAN; EDS, Oxford Instruments, Abingdon, UK).

## 3. Results and Discussion

### 3.1. Optimization of Micro-Beam X-ray Fluorescence Test Conditions for IN718 Alloy

**Figure 3:**
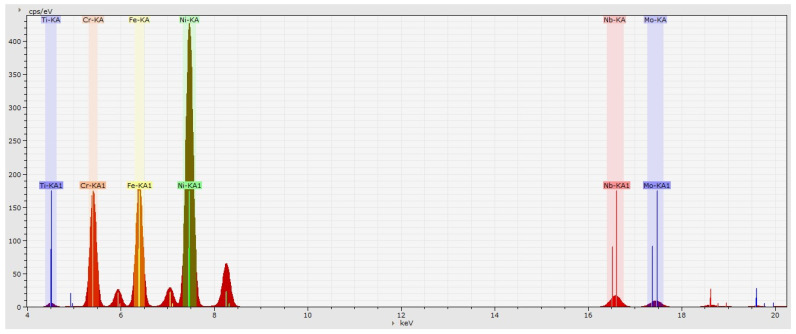
Typical X-ray fluorescence spectrum of Inconel 718 superalloy.

A typical XRF spectrogram of the IN718 superalloy is shown in Figure 3. The spectral peak positions of Ti, Cr, Mn, Fe, and Ni are close to each other, and the Kβ peaks of some elements affect the Kα peaks of other elements. For example, the Kβ peak of Mn (*E* = 6492 eV) is close to the Kα peak of Fe (*E* = 6405 eV), which can cause an apparent higher concentration result for Fe. For Nb, the Kα line (*E* = 16,615 eV) is far away from the spectral positions of the remaining elements and can be completely separated from the Kα line of Mo (*E* = 17,480 eV), and thus it has fewer interferences.

Factors that affect XRF testing mainly include the tube voltage (kV), tube current (mA), pixel time (ms), filters, and vacuum. The operating voltage of the X-ray tube should be increased for heavier elements because of the higher critical excitation potential. The operating voltage is usually set at two to four times the critical excitation voltage. The corresponding excitation voltages for different elements are shown in Table 3. When exciting the K-lines of Cr, Fe, Mo, Nb, and Ti, the tube voltage should be above 40 kV, as employed in this study.

Tube current is also a critical parameter in the testing of μ-XRF, as an insufficient intensity of the characteristic peaks can result from low tube currents. However, increasing the tube current also increases the spectrum background caused by scattering peaks, which may lower the PBR of the characteristic peaks. Therefore, the proper tube current must be chosen by considering both the fluorescence intensity and PBR.

Moreover, if a filter with *d* (cm) thickness is used, the incident light intensity, *I*_0_, will be attenuated to *I*, according to the relationship [38]:

(1) $$I/I_{0}=\exp(-\mu \rho d),$$

where *μ* is the filter mass attenuation coefficient (cm^2^/g) and *ρ* is the material density (g/cm^3^). The absorption characteristics of the filter can be used to reduce the intensity of the primary radiation from the X-ray tube and weaken the intensity of the continuous spectral lines, thereby reducing the scattering background and, in particular, reducing the interference from the target characteristic X-ray spectrum and impurity lines, thus improving the PBR and reducing the dead time of the instrument. In the energy range from 5 to 10 keV, an aluminum filter is suitable to reduce the Rh L-series target line and increase the PBR. Several different thicknesses of Al filters are available for the M4 TORANDO instrument. In this study, a thinner filter was preferred to ensure a high intensity of the characteristic signals.

Pixel time is an important parameter when scanning an area. Extending the pixel time enhances the intensity of elemental peaks and makes it possible to detect elements with low atomic numbers or concentrations. However, a long pixel time means a longer scanning time and a decrease in productivity. In practice, the pixel time needs to be chosen to balance the accuracy and efficiency.

Finally, vacuum conditions are necessary for the detection of light elements to minimize the absorption of fluorescence by air. Additionally, vacuum conditions also prevent primary X-ray scattering from the air. The tests were conducted in a vacuum of 2000 Pa.

In spectroscopic analysis, the PBR is an important basis for assessing the quality of a fluorescence spectrum. PBR is the ratio of the characteristic X-ray peak intensity of an element, *I_p_*, to the background intensity, *I_b_*, and is related to the lower limit of detection (*LLD)*. *LLD* is assumed to be the smallest amount of analyte in a specimen that can be detected in a given analytical context with the 95% confidence level [39]. The relation is as follows:

(2) $$LLD=\frac{2\sqrt{2}\sigma_{B}}{SLP}\approx \frac{3}{SLP}\sqrt{\frac{I_{b}}{t}},$$

where *SLP* is the elementary sensitivity, *I_b_* is the background count under the characteristic X-ray peak, and *t* is the measuring time [40].

To optimize the test parameters for the IN718 superalloy and obtain a relatively optimum PBR for different elements, orthogonal tests were sequentially designed. The orthogonal test method (also called the Taguchi method) is a kind of design method used to study many factors and levels. It conducts tests by selecting a suitable number of representative test cases from many test data, which have evenly dispersed, neat, comparable characteristics [41]. The Taguchi method is suitable for solving the stated problem with the minimum number of trials, as compared with a full factorial design. The experiments were designed according to an orthogonal array to show the effects of each potential primary factor. This method reveals which factors are most effective in achieving the goals and the directions in which these factors should be adjusted to improve the results. The control for achieving the goals will be best obtained by changes in these primary factors in the direction indicated by the analysis [42,43].

To shorten the test time, the voltage and current were pre-tested to determine the level ranges. The voltage was set to 40 kV, 45 kV, and 50 kV levels, and the current was set to 120 μA, 140 μA, and 180 μA. The results showed that the PBR of Nb, Mo, Cr, and Fe increased with the increase of the voltage and the decrease of the current. Therefore, the optimal voltage range fell between 45 kV and 50 kV, and the optimal current should not exceed 120 μA.

Four factors (voltage, current, pixel time, and filters) were numbered from A to D and set at two different levels. In addition to these four factors, two-factor interactions were considered. The factors are presented in Table 4.

The PBR of four alloying elements (Nb, Mo, Cr, and Fe) were employed as test indicators. Table 5 presents the L8(2^7^) orthogonal array and corresponding results.

In order to study the results from the orthogonal tests, the range analysis was utilized in this study. Range analysis is a statistical method to determine the factors’ sensitivity to the experimental result according to the orthogonal experiment. Range is defined as the distance between the extreme values of the data. The greater the range is, the more sensitive the factor is. The calculation process of range analysis is as follows:

(3) $$\delta_{Xm}={\bar{I}}_{Xm}-Y,$$

(4) $$R=\max\left(\delta_{X1},\delta_{X2}\right)-min(\delta_{X1},\delta_{X2}),$$

where ${\bar{I}}_{Xm}$ represents the average value of the experimental results that contain the factor *X* with m level. *Y* denotes the average value of all the test results, and *R* indicates the degree of influence of factor *X*.

The results of the range analysis are shown in Table 6.

The range values reflect the extent to which changes in the level of factors affected the test results. According to Table 6, voltage, filter, and pixel time are the main factors that affected the PBR. The PBR value increased as the operating voltage increased and the pixel time decreased under the experimental conditions. The addition of a 12.5 μm Al filter enhanced the spectra of the testing elements. Taking Nb as an example, the pivotal factor influencing its PBR value was the pixel time, with a PBR range of 3.13. Notably, voltage and current had an inevitable interaction in these tests.

Based on this optimization of the μ-XRF conditions, subsequent test conditions were set to 50 kV voltage, 120 μA current, 100 ms pixel time, and a 20 μm pixel step with a 12.5 μm Al filter to obtain better spectral results.

### 3.2. Quantitative Calibration of Micro-Beam X-ray Fluorescence for IN718 Superalloy

**Figure 4:**
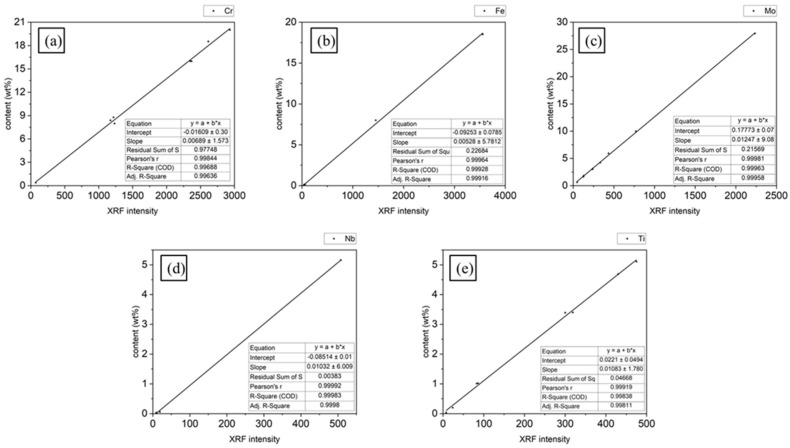
Calibration curves of X-ray fluorescence intensity and reference concentration of nickel-based alloy. (**a**) Cr, (**b**) Fe, (**c**) Mo, (**d**) Nb, and (**e**) Ti.

To achieve quantitative XRF analysis of the nickel-based superalloy, the 11 nickel-based alloy spectrometric reference materials listed in Table 2 were used for area scanning analysis under the optimized test parameters obtained in Section 3.1. The average XRF intensity was linearly fitted to the reference concentration listed in Table 2 to obtain calibration curves, as shown in Figure 4.

Figure 4 shows that the calibration curves for the five alloying elements exhibited excellent linear correlation. Consequently, these elements can be quantitatively characterized in nickel-based superalloys.

### 3.3. Concentration Distribution of Elements and Statistical Analysis

**Figure 5:**
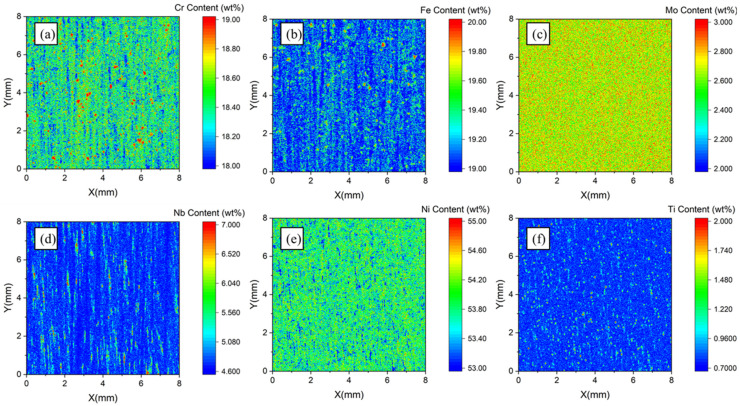
Quantitative distribution results of the S1 test area. (**a**) Cr, (**b**) Fe, (**c**) Mo, (**d**) Nb, (**e**) Ni, and (**f**) Ti.

Area scanning was carried out on the S1 test area using a 20 μm pixel step. A 400 × 400 intensity matrix could be obtained by testing a rectangular area of 8 mm × 8 mm. The XRF intensity matrix was then converted to a concentration matrix using the calibration curves in Figure 4. Following this, two-dimensional distribution maps of the elemental concentration were plotted, as shown in Figure 5.

**Figure 6:**
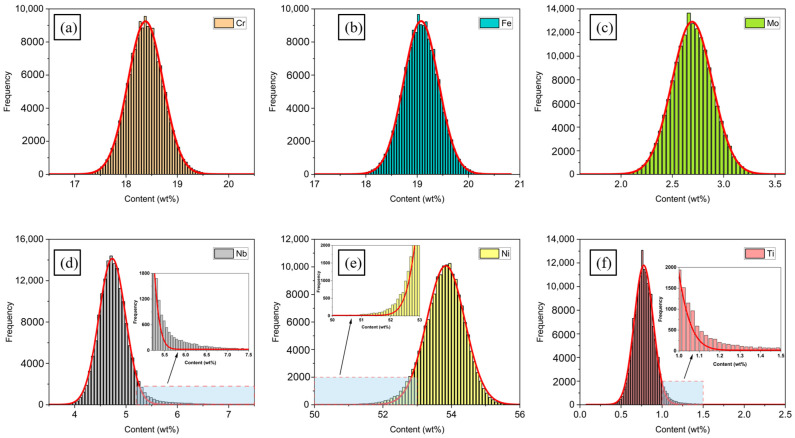
Content–frequency statistic distribution on the entire scanning area for (**a**) Cr, (**b**) Fe, (**c**) Mo, (**d**) Nb, (**e**) Ni, and (**f**) Ti.

In Figure 5, Cr, Fe, Nb, and Ti had obvious positive segregation regions on the S1 test area, where Cr, Fe, and Ti showed punctate segregation while Nb showed banded segregation. Ni mainly presented negative segregation in the test area, where the Nb concentration was often high. The content–frequency statistic distribution of six elements is plotted in Figure 6.

If the distribution of an element is homogeneous, the content–frequency distribution can be described by a Gaussian function. There was a little tailing profile presented on the right side of the curve for Nb and Ti, as shown in Figure 6, which indicated that a large amount of abnormally high concentration values of Nb and Ti existed on the scanning area. Therefore, obvious positive segregation of Nb and Ti took place on the scanning area, as shown in Figure 5. However, the content–frequency distribution of Ni presented an opposite trend to that of Nb and Ti. It was found that a tailing profile occurred on the left side of the content–frequency distribution, which was caused by some lower concentration points of Ni; thus, severe negative segregation of Ni occurred on the scanning area, as shown in Figure 5. To further characterize the degree of segregation in S1, an original position statistical distribution analysis was introduced [44]. The maximum segregation degree, *M*(*x*, *y*), is expressed as the ratio of the element maximum concentration, $C_{max}$, to the average concentration, $\bar{C}$, on the scanning area:

(5) $$M\left(x,y\right)=C_{max}/\bar{C}.$$

The minimum segregation degree is similarly expressed as the ratio of the minimum to the average concentration of the element. The statistic segregation degree of the elements was calculated according to Formula (4):

(6) $$S=(C_{2}-C_{1})/2C_{0}.$$

where *C*_1_ and *C*_2_ are the lower and upper limits of the elemental concentration at the 95% confidence level, and *C*_0_ is the median concentration value calculated from the whole concentration value matrix. Table 7 shows the statistical results of elemental segregation on the scanning area.

**Figure 7:**
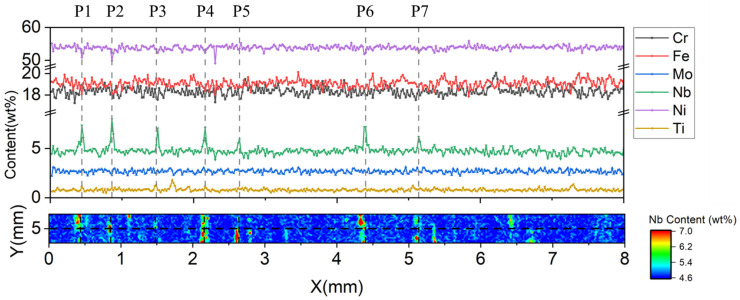
Concentration distributions of different elements at *Y* = 5 mm on the scanning area.

The results for S1 showed that Nb and Ti had higher maximum segregation degrees and standard deviations than the other elements. The maximum segregation degrees of Nb and Ti on the test area were 3.88 and 6.86, and the statistical segregation degrees were 0.15 and 0.35, respectively. The concentration distributions of the six elements along the X direction of the scanning area when the value of coordinate Y was 5 mm are shown in Figure 7. It was found that there were seven obvious high peaks in the line concentration distribution of Nb and similar high concentration peaks also appeared on the line distribution map of Ti. However, negative peaks appeared on the line distribution map of the nickel concentration at the same location where high concentration peaks of Nb occurred. Seven points with significant concentration fluctuations of Nb in Figure 7 were selected for further study. The segregation degree was calculated by dividing the point concentration of the element by the average concentration within the scanning area, and the results are shown in Table 8.

The segregation degrees of Nb and Ti on the seven chosen points all exceeded 1, especially for Nb, which suggests an enrichment of Nb and Ti at these positions. At the position of P2, the segregation degree of Nb was 1.666 and the concentration of Nb was higher than the average concentration of the entire scanning area, by more than 60%. At the positions of P1 and P6, the segregation degree of Nb was also high, reaching 1.535 and 1.504. Due to the positive segregation of Nb and Ti at the selected sites, the concentration of Ni as a matrix constituent was affected, resulting in a lower concentration than the average concentration, with a segregation value below 1. The concentration distribution of Cr, Fe, and Mo was relatively homogeneous, and the segregation degree values were generally close to 1, which indicates there is no obvious segregation trend for these elements.

### 3.4. Correlation between Elemental Segregation and Microstructure Distribution of IN718 Superalloy

**Figure 8:**
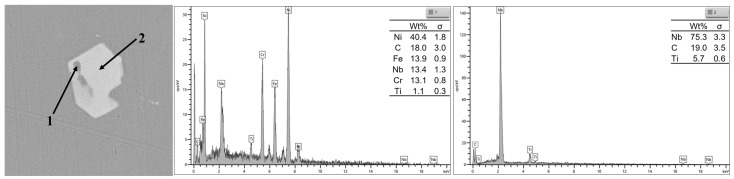
Scanning electron microscopy and energy-dispersive elemental analysis of typical Nb precipitate.

It is widely acknowledged that the elemental distribution of materials is closely related to the composition and distribution of the microstructure. In order to further investigate the reasons for concentration segregation of Nb and Ti in IN718 superalloy, SEM-EDS was applied to characterize the morphology, size, composition, and quantity distribution of precipitates on the same test area of the S1 sample. Typical morphology and composition of precipitates by SEM-EDS are shown in Figure 8. The analysis revealed that the precipitate in the S1 sample exhibited a bright white polygonal morphology, primarily comprising of Nb, Ti, and C. The mass fraction of Nb exceeded 75%, while a small amount of Ti dissolved in the white precipitate phase.

**Figure 9:**
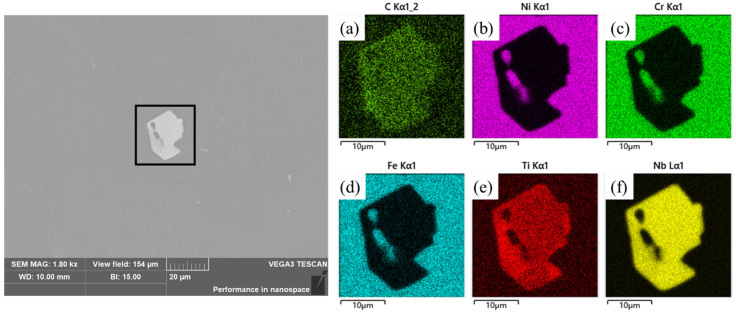
Scanning electron microscopy energy-dispersive spectroscopy mapping results for (**a**) C, (**b**) Ni, (**c**) Cr, (**d**) Fe, (**e**) Ti, and (**f**) Nb.

Figure 9 shows the elemental distribution of the precipitates, and it also indicates that the bright white area was enriched with Nb, Ti, and a small proportion of C, causing a decrease in the concentration of Ni, Cr, and Fe. The results had a good agreement with the segregation distribution characteristic results by μ-XRF.

**Figure 10:**
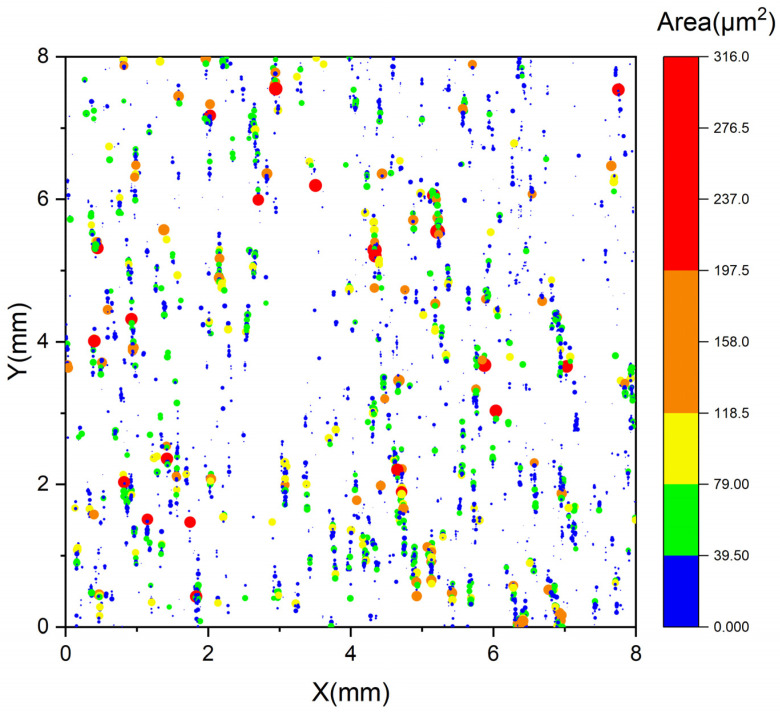
Two-dimensional distributions of Nb precipitates.

The location and distribution of all bright white precipitate particles in the same test area were characterized by SEM-EDS, in combination with μ-XRF scanning analysis. This approach included advanced automatic analysis technology applied to multiple fields of view to ensure all white particles in the scanning area were identified and analyzed. A striped or banded distribution of the bright white precipitate particles in the test area is shown in Figure 10. The distribution pattern closely resembled the concentration distribution of Nb, as shown in Figure 5d. The locations of the Nb precipitates were consistent with the position where positive Nb segregation was observed. Furthermore, it was frequently observed that Ti is present in the Nb precipitates, indicating a tendency for Nb and Ti to have similar segregation behavior, as discussed in Section 3.2. Therefore, it can be concluded that the Nb-containing precipitates led to the concentration segregation of Nb and Ti in the S1 sample.

**Figure 11:**
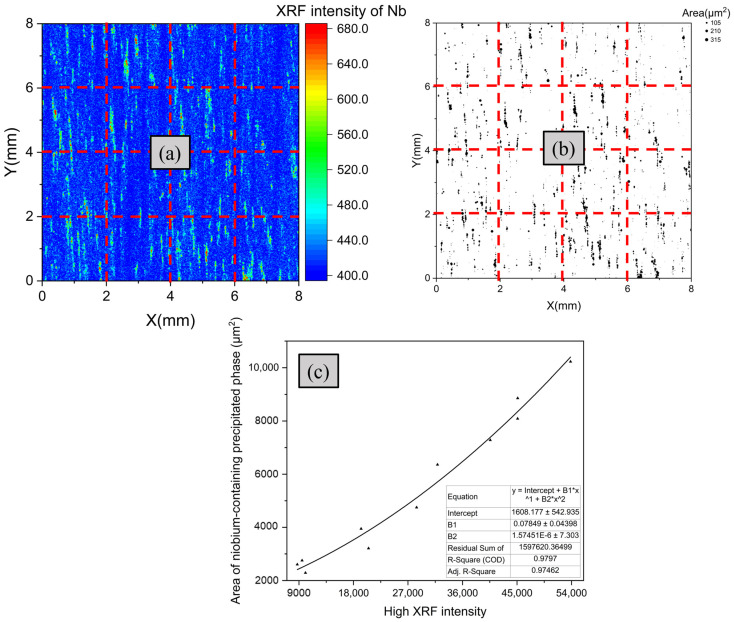
Correlation between the total area of Nb precipitates and the sum of high XRF intensities. (**a**) Distributions of XRF intensities of Nb. (**b**) Distributions of Nb precipitates. (**c**) Fitting curve between high XRF intensity of Nb and the Nb precipitate area.

The influence of precipitates’ size on the XRF intensity of Nb has been studied. To investigate the relationship between the XRF intensity of Nb and Nb precipitates, the 8 mm × 8 mm rectangular area on the test area was separated into 16 rectangular regions of 2 mm × 2 mm, as shown in Figure 11a. An XRF intensity greater than three standard deviations of the mean (>$\bar{x}+3\sigma$) was considered as an intensity threshold value for distinguishing the abnormal signals from all signals of Nb in the test zone. The sums of high XRF intensity from abnormal signals and the Nb precipitate area in each partition were separately counted. Figure 11c displays the binary regression fitting curve of the high XRF intensity and the Nb precipitate area.

The curve shows a strong correlation between high XRF intensity and the area of Nb precipitates. With the increase of the high XRF intensity, the area of Nb precipitates also gradually increased, and the correlation coefficient, R, was close to 0.98. This implies that locations with a high XRF intensity for Nb are likely to indicate the existence of Nb precipitates, with the Nb precipitates being estimated based on the abnormally high intensity of Nb using μ-XRF.

## 4. Conclusions

In this study, μ-XRF spectrometry was used to characterize the elemental segregation of Cr, Fe, Mo, Nb, and Ti in IN718 superalloy. The μ-XRF test conditions for IN718 superalloy were optimized by orthogonal tests. The concentration distributions of Cr, Fe, Mo, Nb, and Ti in IN718 alloy were determined with the optimized testing parameters, and the degree of elemental segregation in different regions of the analytical area was calculated. On this basis, the following conclusions can be drawn:

1. μ-XRF spectroscopy allows simultaneous and quantitative original position statistical distribution analysis of different elements in Inconel 718 alloy.
2. The segregation of Ti and Nb in the sample was obvious, and the statistical segregation degrees of these elements in sample S1 reached 0.35 and 0.15, respectively. The maximum segregation degrees reached 6.86 and 3.88, respectively. Both Nb and Ti showed similar positive segregation, and the matrix elements, such as Ni, showed the opposite segregation behavior.
3. The distributions of Nb concentration and Nb precipitates were strongly correlated. Severe segregation of Nb and Ti elements occurred where Nb-containing precipitates had accumulated.
4. A correlation curve between high XRF intensities of Nb and the area of Nb precipitates was established. Locations with an abnormally high XRF intensity for Nb will likely indicate the presence of Nb precipitates, and the area of Nb precipitates can be calculated by the abnormally high intensity of Nb based on μ-XRF.
5. This study investigated the potential for characterizing material microstructures through μ-XRF, which is anticipated to allow for efficient and non-destructive analysis in the future.

## Figures and Tables

**Table 1:** Chemical composition of S1 (m/m %).

Cr	Fe	Nb	Mo	Ti	Al	Mn	Ni
17.61	18.81	4.97	2.96	0.94	0.55	0.08	Balance

**Table 2:** Chemical compositions of the nickel alloy spectrometric reference materials used in this work (m/m %).

No.	Fe	Cr	Mo	Co	W	Ti	Zr	Nb	Ta
24 × 7201A	0.09	15.99	3.01	14.79	1.29	5.11	0.0432	/	/
IARM277	0.16	14.35	4.22	14.5	0.047	3.4	0.01	0.034	/
IARM325	0.07	18.52	9.98	10.46	0.03	3.16	0.002	0.007	0.003
IMZ180	0.073	7.98	5.93	9.95	/	1.02	0.075	0.025	4.26
IMZ182	/	8.63	3.1	13.52	/	4.69	0.031	/	/
IMZ183A	0.063	15.99	1.63	8.23	2.62	3.39	0.033	0.747	1.56
IMZ187	0.053	8.78	1.82	9.7	6.93	2.31	0.029	0.004	3.79
IMZ202	/	8.39	0.63	10.02	10.04	1.01	0.031	0.028	3.25
BS718D	18.51	18.32	3.00	0.368	0.049	0.93	/	5.16	/
SBS35503-2017	8.00	20.00	15.90	0.055	6.00	0.20	/	/	/
SBS35504-2017	5.94	0.375	27.94	1.70	0.096	0.034	0.016	0.034	/

**Table 3:** Excitation voltages for different elements.

Tube Voltage/kV	K-Series Spectral Line	L-Series Spectral Line
60	Fe-Ba	Sm-U
50	Cr-Mn	Pr-Nd
40	Ti-V	Cs-Ce
30	Ca-Sc	Sb-I
24	Be-K	Ca-Sn

**Table 4:** Process factors and their levels.

Factors	Unit	Level	
		Level 1	Level 2
Voltage (A)	kV	48 (1)	50 (2)
Current (B)	μA	100 (1)	120 (2)
Pixel time (C)	ms	100 (1)	150 (2)
Filter (D)	-	12.5 μm Al (1)	no treatment (2)
Interaction A × B	-	1	2
Interaction A × C	-	1	2

**Table 5:** Orthogonal array and results.

No.	Designation	Interaction A × B	Interaction A × C	Results			
				Nb PBR	Mo PBR	Cr PBR	Fe PBR
1	A1B1C1D1	1	1	75.6	45.3	197.3	164.4
2	A1B1C2D2	1	2	69	42.4	193.4	155.6
3	A1B2C1D2	2	1	70.1	42.4	191	154.3
4	A1B2C2D1	2	2	69.6	42.5	197.7	164.2
5	A2B1C1D2	2	2	73.4	44.1	191.9	155
6	A2B1C2D1	2	1	73.5	45	200.2	167.1
7	A2B2C1D1	1	2	77.4	46.2	197.6	165.4
8	A2B2C2D2	1	1	71.9	44	191.8	155.6

**Table 6:** Range analysis of the orthogonal test.

Factors	Nb PBR	Mo PBR	Cr PBR	Fe PBR
A Voltage/(kV)	2.98	1.68	0.52	1.15
B Current/(μA)	0.63	0.42	1.17	0.65
C Pixel time/(ms)	3.13	1.03	1.32	0.85
D Filter	2.93	1.53	6.18	10.15
Interaction A × B	1.83	0.97	0.17	0.10
Interaction A × C	0.43	0.37	0.08	0.30

**Table 7:** Statistical results of elemental segregation of S1 on the scanning area.

Element	Maximum Value (m/m %)	Minimum Value (m/m %)	Average Value (m/m %)	Maximum Segregation Degree	Minimum Segregation Degree	Standard Deviation (m/m %)	RSD/%	Statistical Segregation Degree
Cr	20.5	13.69	18.38	1.12	0.74	0.36	1.96	0.04
Fe	20.78	13.63	19.07	1.09	0.71	0.365	1.91	0.04
Mo	3.62	1.58	2.69	1.35	0.59	0.199	7.39	0.15
Nb	18.63	2.94	4.8	3.88	0.61	0.091	9.12	0.15
Ni	56.25	36.12	53.77	1.05	0.67	0.702	1.31	0.02
Ti	5.46	0.11	0.8	6.86	0.14	0.168	21.2	0.35

**Table 8:** Segregation degree of different elements from P1 to P7 at *Y* = 5 mm.

Segregation Degree	Cr	Fe	Mo	Nb	Ni	Ti
P1	1.030	0.947	1.038	1.535	0.946	1.437
P2	1.003	0.955	0.850	1.666	0.927	1.257
P3	0.968	1.026	0.921	1.113	0.994	1.633
P4	0.972	0.965	1.025	1.486	0.973	1.480
P5	0.959	0.989	1.015	1.240	0.997	1.096
P6	0.978	0.980	0.983	1.504	0.973	1.162
P7	1.004	0.983	1.000	1.294	0.975	1.050

## Data Availability

All data included in this study are available upon request by contact with the corresponding author.
